# Pheromone sensing in *Drosophila* requires support cell-expressed Osiris 8

**DOI:** 10.1186/s12915-022-01425-w

**Published:** 2022-10-11

**Authors:** Marta Scalzotto, Renny Ng, Steeve Cruchet, Michael Saina, Jan Armida, Chih-Ying Su, Richard Benton

**Affiliations:** 1 Center for Integrative Genomics, Faculty of Biology and Medicine, University of Lausanne, CH-1015 Lausanne, Switzerland; 2 Neurobiology Section, Division of Biological Sciences, University of California San Diego, La Jolla, CA 92093 USA

**Keywords:** Olfactory subsystem, *Drosophila melanogaster*, Comparative transcriptomics, Sensory neuron, Support cell, Pheromone detection, Osiris 8

## Abstract

**Background:**

The nose of most animals comprises multiple sensory subsystems, which are defined by the expression of different olfactory receptor families. *Drosophila melanogaster* antennae contain two morphologically and functionally distinct subsystems that express odorant receptors (Ors) or ionotropic receptors (Irs). Although these receptors have been thoroughly characterized in this species, the subsystem-specific expression and roles of other genes are much less well-understood.

**Results:**

Here we generate subsystem-specific transcriptomic datasets to identify hundreds of genes, encoding diverse protein classes, that are selectively enriched in either Or or Ir subsystems. Using single-cell antennal transcriptomic data and RNA in situ hybridization, we find that most neuronal genes—other than sensory receptor genes—are broadly expressed within the subsystems. By contrast, we identify many non-neuronal genes that exhibit highly selective expression, revealing substantial molecular heterogeneity in the non-neuronal cellular components of the olfactory subsystems. We characterize one Or subsystem-specific non-neuronal molecule, Osiris 8 (Osi8), a conserved member of a large, insect-specific family of transmembrane proteins. Osi8 is expressed in the membranes of tormogen support cells of pheromone-sensing trichoid sensilla. Loss of Osi8 does not have obvious impact on trichoid sensillar development or basal neuronal activity, but abolishes high sensitivity responses to pheromone ligands.

**Conclusions:**

This work identifies a new protein required for insect pheromone detection, emphasizes the importance of support cells in neuronal sensory functions, and provides a resource for future characterization of other olfactory subsystem-specific genes.

**Supplementary Information:**

The online version contains supplementary material available at 10.1186/s12915-022-01425-w.

## Background

To fulfil the formidable task of detecting and discriminating many diverse chemical signals in the external world, animal olfactory systems contain tens to thousands of distinct olfactory sensory neuron (OSN) populations. In both vertebrates and insects, each neuronal population is distinguished by the expression of a specific olfactory receptor (or, occasionally, receptors) and their innervation pattern in the brain [1, 2]. In most species, olfactory systems are also characterized by the gross-level organization of different sets of OSNs into structurally and functionally distinct “subsystems.” In rodents, for example, the main olfactory epithelium and vomeronasal organ express different receptor families and have distinct (though not exclusive) roles in detecting environmental odors and pheromones, respectively [3–6].

**Fig. 1:**
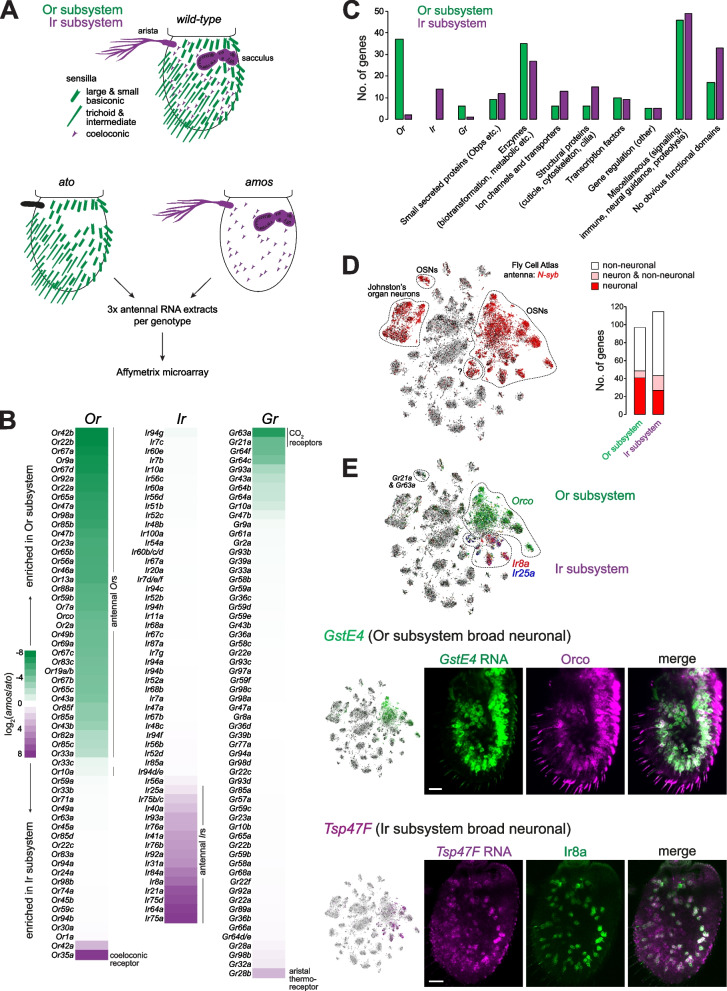
A transcriptomic screen for olfactory subsystem-specific genes. **A** Top: schematic of the *D. melanogaster* antennal olfactory subsystems. Bottom: schematic of the comparative antennal transcriptomics experiment of *ato* mutant (*ato*^*1*^*/Df(3R)p13*) and *amos* mutant (*amos*^*3*^) animals. **B** Heatmap showing differential expression of chemosensory receptor gene families (*odorant receptor* (*Or*), *ionotropic receptor* (*Ir*), and *gustatory receptor* (*Gr*)) in *ato* and *amos* antennal transcriptomes. The enrichment (or non-enrichment) of genes is as expected in all cases (see “Results”), with a few exceptions: (i) *Or33c* and *Or42a* are thought to be expressed specifically in the maxillary palp [17, 28]; however, *Or33c* was previously detected in the antenna by qRT-PCR [16] and *Or42a* transcripts have been detected in some *Orco*-negative neurons in the Fly Cell Atlas [29]. *Ir68a* encodes a hygroreceptor that acts in sacculus neurons [30], although transcripts of this gene appear to be expressed at low levels [31]. **C** Bar chart comparing the classification of Ir and Or subsystem-enriched genes into the indicated categories (see Additional file 4: Table S1). **D** Left: *t*-distributed stochastic neighbor embedding (tSNE) representation of RNA-seq datasets from individual antennal cells from the Fly Cell Atlas (10× stringent dataset in this and all subsequent figures) [29], colored for expression of the neuronal marker *N-syb*, which is expressed in OSNs, Johnston’s organ auditory neurons, and a neuron population of unknown identity (marked “?”; these also express the mechanoreceptor *NompC*, suggesting that they are auditory/mechanosensory, rather than olfactory). Right: bar chart of the expression of olfactory subsystem-enriched genes in neuronal and non-neuronal antennal cell populations (see Additional file 4: Table S1). **E** Top: tSNE plot of antennal single-cell transcriptomes colored for expression of Or and Ir co-receptors, which demarcate the two olfactory subsystems (although *Ir25a* is expressed at low levels across both subsystems [26, 32]). The *Gr21a/Gr63a* cell cluster is also indicated; although these do not express *Orco*, they are considered part of the Or subsystem. Bottom left: tSNE plots colored for expression of the Or subsystem-enriched *GstE4* and Ir subsystem-enriched *Tsp47F*. Bottom right: combined RNA FISH and immunofluorescence for *GstE4* and Orco (top) or *Tsp47F* and Ir8a (bottom) on whole-mount antennae of control (*w*^*1118*^) animals confirming the broad, subsystem-enriched neuronal expression of these genes. Scale bars, 20 μm

Insects also possess olfactory subsystems, which have been best-described in *Drosophila melanogaster* [7–10]. The main *D. melanogaster* olfactory organ is the antenna (Fig. 1A), a head appendage covered with several hundred sensory sensilla [11, 12]. Each sensillum constitutes a porous cuticular hair housing the ciliated dendrites of 1-4 OSNs, whose somas are flanked by non-neuronal support cells [13]. Antennal OSNs can be categorized into two subsystems, defined by their expression of members of distinct olfactory receptor repertoires: the odorant receptors (Ors) [14–18] and the ionotropic receptors (Irs) [10, 19]. Receptors from both families function as odor-gated ion channels, composed of ligand-specific “tuning” receptors and one or more broadly expressed co-receptors: Orco for Ors; Ir8a or Ir25a and Ir76b for Irs [20–27]. Although the Or and Ir subsystems share a similar overall organization and are intermingled within the antenna, they display a number of important differences.

During development, the Or and Ir subsystems are specified by distinct transcription factors—*a*bsent *m*ultidendritic neurons and *o*lfactory *s*ensilla (Amos) and *Ato*nal (Ato), respectively—which determine the fate of lineages derived from sensory organ precursors in the developing larval antennal imaginal disk [33–35]. Amos and Ato induce the expression of downstream genes required to form the corresponding olfactory subsystem, encompassing neurons, non-neuronal support cells, and other structural elements. Morphologically, the sensilla in the Or subsystem comprise three main classes (basiconic, trichoid, and intermediate) while Ir subsystem OSNs are housed in coeloconic sensilla [11, 12]. Some Ir neurons are found in hygrosensory or thermosensory sensilla in the sacculus (an internal multi-chambered pocket) and arista (a long cuticular projection) (Fig. 1A) [30, 36–39]. Different sensillar classes exhibit several distinct structural features, including hair length and diameter, pore size and number, and the sensory cilia morphology of OSNs and the number of support cells [12, 40–42].

The response properties of Or and Ir OSNs are also distinctive, encompassing different odor specificities, sensitivities, and temporal dynamics [10, 43, 44]. Much effort has focused on addressing how Ors or Irs define odor-evoked activity, demonstrating that OSN signaling properties are largely determined by the corresponding tuning receptor [26, 45–49]. However, support cells are also likely to have several important contributions to olfactory detection. During development, the non-neuronal cells have roles in secreting and shaping the cuticular structure of olfactory hairs [13, 50]. In mature antennae, support cells are thought to form an isolated biochemical microenvironment for the OSNs within a given sensillum, through their control of the ionic composition of lymph fluid and the secretion of odorant-degrading enzymes and odorant-binding proteins (Obps) [13, 51]. Obps have various perireceptor roles in modulating olfactory responses [13, 52–55]. For example, the Obp Lush is secreted by Or subsystem trichoid sensilla support cells and is critical for the responses of Or67d neurons to their cognate pheromone ligand [56, 57]. The differential expression of this and other Obps in the Or and Ir subsystems [54] raises the question of the existence of other types of olfactory subsystem-specific support cell proteins that contribute to olfactory detection properties.

## Results

### A transcriptomic screen for olfactory subsystem-specific genes

To identify subsystem-specific molecules, encompassing those expressed in neuronal and/or non-neuronal cells, we performed comparative transcriptomics of antennae from animals mutant for either *amos* or *ato*, which selectively lack the Or or Ir subsystems, respectively (Fig. 1A) [33–35, 58]. This strategy was designed to facilitate sensitive identification of differentially expressed genes between the two subsystems, complementary to previously described independent comparisons of the antennal transcriptomes of these mutants against controls [59, 60]. We isolated RNA from *amos* or *ato* antennal olfactory segments (in three biological replicates) and hybridized these samples to *D. melanogaster* microarrays (Fig. 1A).

Comparison of transcript levels for positive control genes (i.e., *Or*s and *Ir*s) validated the selectivity and sensitivity of this screen (Fig. 1B and Additional file 4: Table S1): with very rare exceptions, all transcripts for antennal *Or*s (but not those expressed in other olfactory organs [11], or the exceptional Ir subsystem-expressed *Or35a* [47]) were detected at significantly lower levels in *amos* compared to *ato* antennae (Fig. 1B and Additional file 4: Table S1). Conversely, transcripts for essentially all antennal Irs (but not family members expressed in non-olfactory organs [31, 61–63]) were expressed at lower levels in *ato* antennae compared to *amos* mutants. We further analyzed members of the “*gustatory receptor*” (*Gr*) gene family: most *Gr*s are expressed in contact chemosensory organs [64] and, consistently, are not differentially expressed in these olfactory subsystems (Fig. 1B). However, a few *Gr*s have sensory functions in the antenna: *Gr21a* and *Gr63a* encode subunits of a carbon dioxide receptor expressed in basiconic sensilla [65, 66] and their transcripts were enriched, as expected, in the Or subsystem (Fig. 1B). By contrast, transcripts for *Gr28b.d* (encoding an aristal-expressed thermoreceptor [38]) were expressed predominantly in the Ir subsystem (Fig. 1B). Several other *Gr*s were mildly enriched in one or other subsystem but their endogenous expression and function (if any) is unclear (Fig. 1B) [67].

To characterize other differentially expressed genes of the olfactory subsystems, we first investigated whether those displaying >2-fold higher expression in either subsystem were enriched for particular Gene Ontology (GO) terms (Additional file 9: Data S1) [68]. Almost all significantly enriched terms reflected the differential expression of the olfactory receptor gene families (e.g., Or subsystem: GO:0007608 sensory perception of smell (FDR q-value 1.11E−13); Ir subsystem: GO:0004970 ionotropic glutamate receptor activity (FDR q-value 6.24E−06)), suggesting that there are few other broad categories of genes that distinguish these subsystems. We did note that the Or subsystem was enriched for genes encoding proteins with glutathione transferase activity (GO:0004364, FDR q-value 1.86E−07). Such enzymes have putative roles in odor degradation [69], and this enrichment might reflect differences in the types of chemical ligands detected by each subsystem [10].

We subsequently focused on genes displaying an expression difference between *amos* and *ato* antennae of >4-fold. This threshold captured the most enriched 177 and 180 genes of the Or and Ir subsystems, respectively—including all of the known chemosensory receptors—and facilitated manual curation and prioritization (Additional file 4: Table S1). Through protein domain and BLAST analyses, we assigned these genes to distinct categories (Fig. 1C). These classes include small, secreted proteins, notably Obps, such as the Or subsystem-expressed Lush [56] and the Ir subsystem-enriched Obp59a, which functions in hygrosensation in the sacculus [54, 70]. Many differentially expressed genes encode enzymes, including those potentially involved in odorant degradation and intracellular metabolism. A number of genes encode ion channels or transporters, some of which have known roles in olfactory signaling. For example, the Ir subsystem-specific Ammonium transporter (Amt) is a non-canonical olfactory receptor underlying ammonia detection in coeloconic and sacculus neurons [60, 71], while the Or subsystem is enriched for the phospholipid flippase/transporter ATPase 8B, which is required for olfactory sensitivity in several classes of basiconic and trichoid OSNs [72, 73]. Several genes encode proteins with probable roles in sensillum construction and/or maintenance, encompassing OSN cilia morphogenesis (e.g., CG45105, an ortholog of human SDCCAG8, which is implicated in ciliopathies [74]) and cuticle formation (e.g., *Vajk3* [75]); some of these may contribute to the distinctive ultrastructural properties of different sensillar classes [12, 40, 41]. Differentially expressed transcription factor genes include *unplugged*, which is required for correct receptor expression and axon targeting in several Or OSN populations [76]. Diverse additional functional categories were represented, including post-translational gene regulation, neural guidance, immune signaling, and proteolysis, while many genes encode proteins with no obvious known domains. Notably, more than half of the differentially expressed genes are functionally uncharacterized, either in or beyond the olfactory system.

### Diverse cell type and breadth of expression of novel olfactory subsystem-enriched genes

To further characterize these differentially expressed genes, we first took advantage of the single-cell RNA-sequencing (scRNA-seq) dataset of the antenna, generated as part of the Fly Cell Atlas [29], to obtain insights into their cellular-level expression patterns. This dataset encompasses the nuclear transcriptomes of cells from all segments of the antenna, which have been clustered and annotated into several dozen neuronal and non-neuronal cell types ([29] and Fig. 1D). We first asked whether the olfactory subsystem-enriched genes were predominantly expressed in neurons or non-neuronal cells by inspection of their expression pattern across cell clusters. We used *N-synaptobrevin* (*N-syb*) as a marker of all neuronal cell types (Fig. 1D) and found that other subsystem-enriched genes (excluding chemosensory receptors) could be neuronal, non-neuronal, or both (Fig. 1D and Additional file 4: Table S1). In both Or and Ir subsystems, the majority of detected genes was expressed in non-neuronal cells.

When we used these scRNA-seq data to survey the breadth of expression of each gene, we found patterns ranging from a single cluster to very broad expression across many cell classes of the Ir or Or subsystems (Additional file 4: Table S1). Notably, the vast majority of neuronal genes are broadly expressed, in contrast to the *Or*s and *Ir*s which are restricted to a single cluster [29]. We confirmed these transcriptomic data by RNA fluorescence in situ hybridization (FISH) for the Or subsystem-enriched *Glutathione S transferase E4* (*GstE4*) and the Ir subsystem-enriched *Tetraspanin 47F* (*Tsp47F*), which are co-expressed with the Orco and Ir8a co-receptors, respectively (Fig. 1E). Although we cannot exclude the possibility that lowly expressed neuron subtype-specific genes are not represented in the scRNA-seq datasets, our data reinforce previous conclusions that the olfactory receptor is the main, and perhaps only, determinant of tuning specificity of most individual neuron classes [26, 45–49]. Other neuronal, subsystem-specific genes identified here may have broader functions in defining these neurons’ signaling, metabolic, and/or morphological properties.

**Fig. 2:**
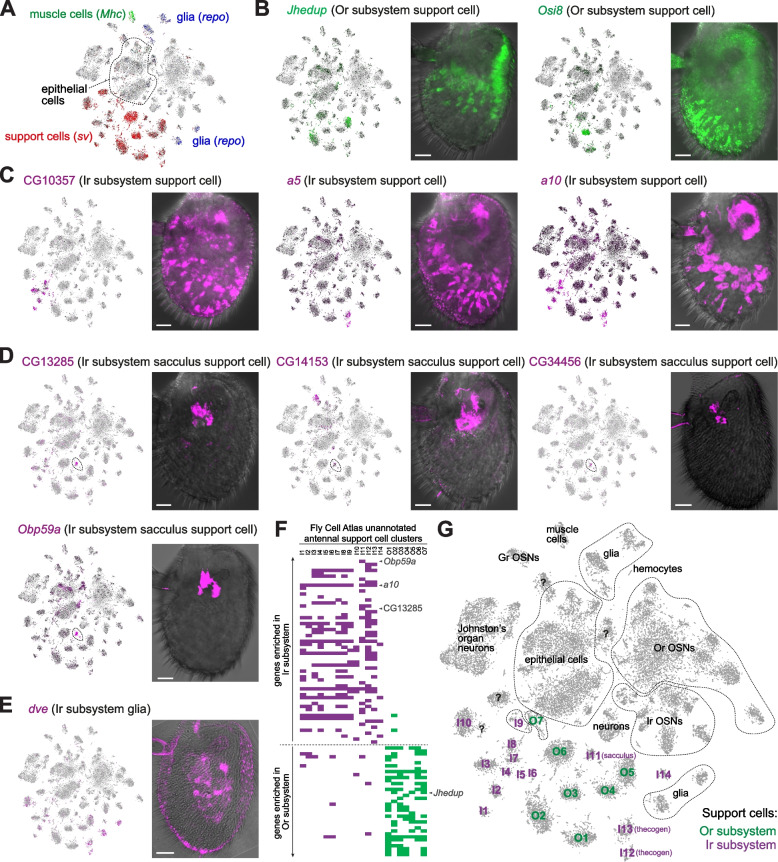
Diverse breadth and spatial location of non-neuronal olfactory subsystem-enriched genes. **A** tSNE plot of antennal single-cell transcriptomes highlighting non-neuronal classes in the antenna, as defined by expression of the indicated marker genes (based upon [29]). **B–E** tSNE plots of antennal single-cell transcriptomes and RNA FISH on whole-mount antennae of wild-type (*Canton-S*) animals illustrating non-neuronal expression patterns of various (**B**) Or subsystem-enriched and (**C–E**) Ir subsystem-enriched genes. Expression of *a5* and *a10* was previously described, but not related specifically to the Ir subsystem [80, 81]; the *Jhedup* RNA FISH expression pattern is consistent with observations of a transgenic promoter reporter for this gene [82]. The sacculus-specific *Obp59a* expression pattern—as previously described [54, 70]—is shown for comparison with novel, sacculus support cell-expressed genes. The bright-field channel is overlaid to reveal cuticle morphology; occasional fluorescence signal within sensillar hairs is likely artefactual. Scale bars, 20 μm. **F** Demarcation of antennal unannotated support cell clusters (I1-14, O1-7) through their expression of Ir and Or subsystem-enriched genes (shaded magenta and green boxes, respectively). Select genes illustrated in **B–D** are highlighted; see Additional file 5: Table S2 for the full dataset). Although expression was assessed qualitatively, not quantitatively, support cells could easily be categorized to each subsystem through their “fingerprint” of subsystem-specific gene expression. **G** tSNE plot of antennal single-cell transcriptomes in which antennal support cell clusters are assigned to the Or and Ir subsystems based upon their expression of subsystem-enriched genes. I11 likely represent sacculus support cells (see “Results” and **D**). Other antennal cell classes are also indicated (based upon [29]). *sv*-positive clusters (see **A**) labeled with “?” (e.g., near I10) were not reliably marked by Or or Ir subsystem-specific genes; these may represent support cells in the Johnston’s organ (or first antennal segment). As Johnston’s organ development is *ato*-dependent [83], we cannot exclude that there are shared markers between support cells of these two segments and that some of the “Ir subsystem” support cell clusters correspond to cells of this auditory organ

We next focused on the non-neuronal genes enriched in either olfactory subsystem. Many non-neuronal cells in the antenna develop independently of the cell lineages of the Or and Ir subsystems, including epithelial cells, muscle cells (which are probably located only within the proximal, non-olfactory antennal segments [77, 78]), and a subset of glia (which migrate from the central brain during development [79]) (Fig. 2A). A distinct set of antennal glia derive from the *ato*-dependent lineages [79], although it is likely that they contribute to the function of both olfactory subsystems. Non-neuronal cells of the olfactory subsystems—known as support (or auxiliary) cells—derive from the same developmental lineages that produce the OSNs within a given sensillum and comprise several classes: trichogen, thecogen, tormogen (described further below). These cell types are represented by ~20 cell clusters in the antennal atlas, globally demarcated by expression of the transcription factor gene *shaven* (*sv*) (Fig. 2A) [29]. The olfactory subsystem-specific genes that we identified display diverse breadth of expression in these cell types (Additional file 4: Table S1), several of which could be validated through RNA FISH (Fig. 2B–E). Some are expressed in multiple cell clusters, such as *Juvenile hormone esterase duplication* (*Jhedup*) in the Or subsystem (Fig. 2B) and CG10357, *a5* and *a10* (all of unknown function) in the Ir subsystem (Fig. 2C). Others are prominently expressed in only one cluster, including the Or subsystem-specific *Osiris 8* (*Osi8*) (Fig. 2B) and the Ir subsystem-specific CG14153, CG13285, and CG34456, which are found in support cells around the sacculus (Fig. 2D). Beyond support cells, we detected glial expression for the Ir subsystem gene *defective proventriculus* (*dve*) (Fig. 2E).

We noted that the sacculus-expressed genes were detectable in the same cluster in the Fly Cell Atlas—which was also marked by the previously characterized *Obp59a* [54, 70] (Fig. 2D)—allowing us to annotate this cluster (arbitrarily named “I11”; Additional file 5: Table S2) as “sacculus support cell” (Fig. 2F,G). We extended this approach by systematically noting the support cell clusters expressing Or and Ir subsystem-enriched genes (Additional file 5: Table S2), which enabled demarcation of essentially all clusters as corresponding to one or other olfactory subsystem (Fig. 2F,G). We were unable to further define support cell types—although I12 and I13 are likely to be thecogen cells (Additional file 5: Table S2)—as many molecular markers are expressed in multiple cell clusters (Fig. 2F,G and Additional file 5: Table S2) and/or multiple sensillum classes (e.g., *Obps* [54]).

### Expression of Osi8 in trichoid sensilla tormogen support cells

**Fig. 3:**
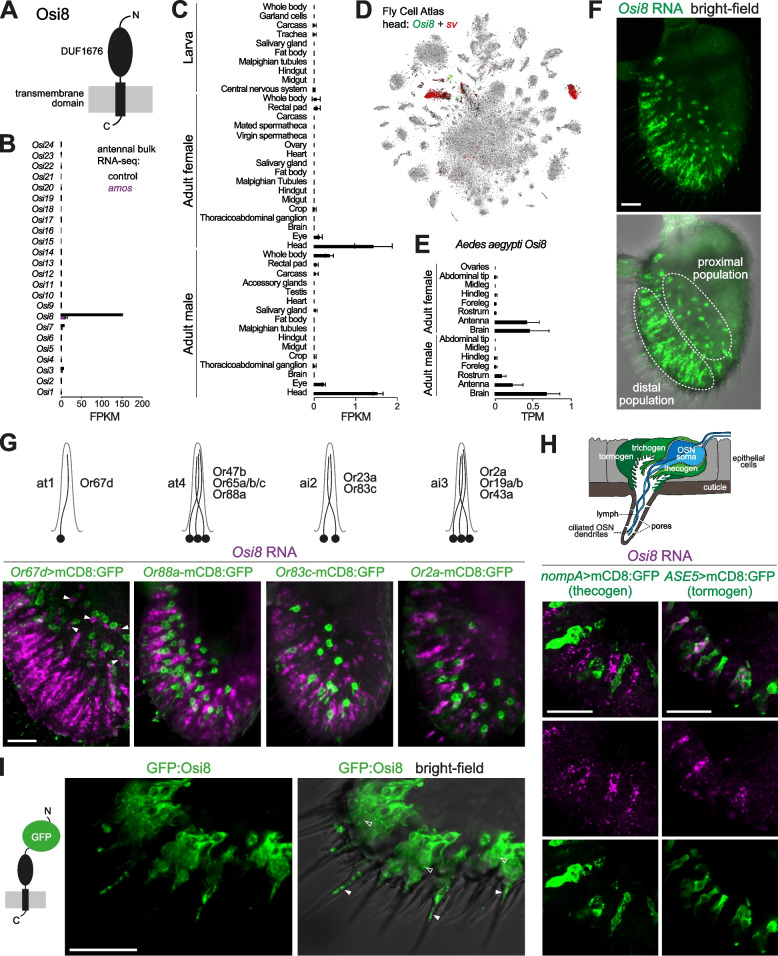
Expression of *Osi8* in the antenna. **A** Schematic of the protein domain structure of Osi8. **B** Histogram of expression levels of *Osi* family members in antennae of control (*w*^*1118*^) and *amos* mutant (*amos*^*3*^) animals, determined by bulk RNA-seq. Mean values ±SD of fragments per kilobase of transcript per million mapped reads (FPKM) are plotted; *n* = 3 biological replicates. Note that *Osi10* values represent the combined counts of *Osi10a* and *Osi10b*. **C** Histogram of *Osi8* expression levels in the indicated *D. melanogaster* tissues determined by bulk RNA-seq; mean FPKM values ±SD are plotted (*n* = 2–3 biological replicates; data are from the Fly Atlas 2.0 [88]). **D** tSNE plot of whole head single-cell transcriptomes (Fly Cell Atlas 10× stringent dataset [29]) highlighting the selective detection of *Osi8* within a subset of *sv*-positive cells (most of which are likely to be antennal support cells (Fig. 2A)). **E** Histogram of expression levels of the *Aedes aegypti Osi8* ortholog (AAEL004275) in the indicated tissues determined by bulk RNA-seq; mean values ±SD of transcripts per kilobase million mapped reads (TPM) are plotted (*n* = 3–8 biological replicates; data are from [89]). **F ***Osi8* RNA FISH on a whole-mount antenna of a control (*w*^*1118*^) animal; the bright-field channel is overlaid on the lower image to reveal cuticle morphology. Morphologically distinct proximal and distal populations of *Osi8* RNA-expressing cells are indicated (see “Results”). Scale bar, 20 μm. **G** Top: schematic of the neuronal composition of the antennal trichoid (at) and antennal intermediate (ai) sensillar classes. Bottom: *Osi8* RNA FISH and GFP immunofluorescence in whole-mount antennae of animals in which the distributions of the different sensillar classes are revealed with a representative Or neuron transgenic reporter. In the left-hand image, the arrowheads indicate *Osi8*-expressing cells that are found in close proximity to Or67d neurons (see “Results”). Genotypes (left-to-right): *UAS-mCD8:GFP/+;Or67d*^*Gal4#1*^*/+*, *Or88a-mCD8:GFP*, *Or83c-mCD8:GFP*, *Or2a-mCD8:GFP*. Scale bar, 20 μm. **H** Top: schematic of an olfactory sensillum, illustrating the main cell types and other anatomical features. Bottom: *Osi8* RNA FISH and GFP immunofluorescence on antennal cryosections of animals in which the tormogen (*UAS-mCD8:GFP;ASE5-Gal4*) or thecogen (*nompA-Gal4;UAS-mCD8:GFP*) support cells are labeled. Scale bars, 20 μm. **I** Immunofluorescence for GFP on antennal cryosections of *Osi8-Gal4/+;UAS-SS:EGFP:Osi8/+* animals; the bright-field channel is overlaid on the right-hand image to reveal cuticle morphology. The open arrowheads point to prominent intracellular puncta of GFP signal. The filled arrowheads point to GFP signal within the lumen of the proximal region of trichoid sensillar hairs, which may represent extracellular vacuoles (see “Results”). Scale bar, 20 μm

We subsequently focused on characterizing the Or subsystem gene *Osi8*, a member of a family of 24 *Osi* genes that each encode an N-terminal signal peptide, a domain of unknown function (DUF1676), followed by a presumed transmembrane domain (Fig. 3A). This family appears to be largely insect-specific, with syntenically arranged orthologs of most members present in diverse insect orders and, with rare exceptions [84], no related genes in the genomes of non-insect Arthropoda or other invertebrates [84–86]. Notably, of the analyzed insect species, *Osi8* is absent in only the human body louse *Pediculus humanus* [86], which also has a greatly reduced *Or* repertoire compared to many other insects [87].

*Osi8* was the only subsystem-enriched member of this family (Additional file 4: Table S1). To confirm and extend this observation, we quantified all *Osi* gene transcripts in a bulk RNA-seq dataset of adult antennae from control and *amos* mutant animals (Fig. 3B). *Osi8* is expressed at >10-fold the level of any other *Osi* gene. Moreover, consistent with our microarray analysis, *Osi8* transcripts are almost completely lost in *amos* mutants (Fig. 3B). Using the Fly Atlas 2.0 [88], we further examined *Osi8* expression in RNA-seq datasets across diverse *D. melanogaster* tissues of both adults and larvae, and found the highest expression levels in adult heads of both males and females (Fig. 3C). This signal is most likely due to the antennal expression of *Osi8* because, within the Fly Cell Atlas whole head dataset [29], transcripts of this gene were detected in only a very small subset of *sv*-positive cells, which presumably correspond to the antennal support cell population (Fig. 3D). Finally, analysis of tissue-specific RNA-seq datasets in the mosquito *Aedes aegypti* [89] revealed high expression of the *Osi8* ortholog (AAEL004275) in the antenna (Fig. 3E). However, transcripts could also be detected in the mosquito brain (Fig. 3E), suggesting additional roles for the gene in this species.

**Fig. 4:**
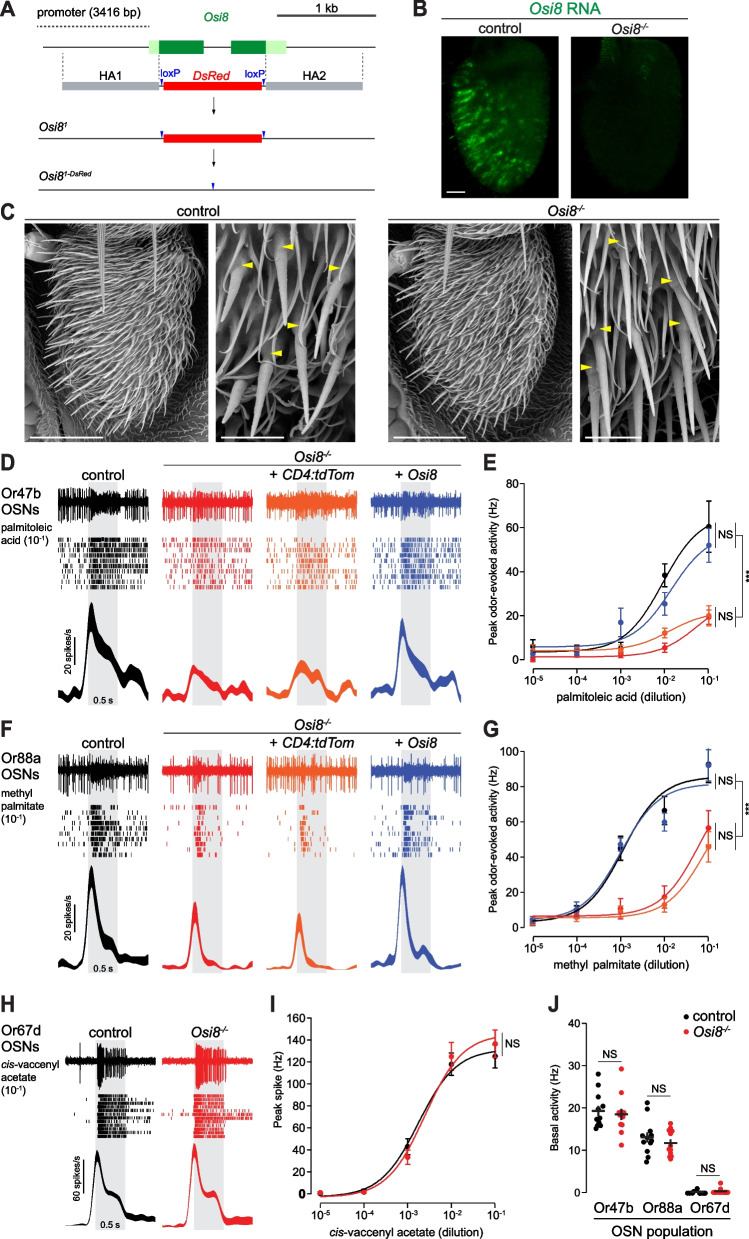
Functional analysis of *Osi8* reveals a selective role in pheromone sensing. **A** Schematic of the generation of the *Osi8*^*1*^ mutant. *Osi8* exons and UTRs are shaded dark and light green, respectively. **B*** Osi8* RNA FISH on whole-mount antennae from control (*w*^*1118*^) and *Osi8*^*1*^ mutant animals. Scale bar, 20 μm. **C** Scanning electron micrographs of antennae from control (*w*^*1118*^) and *Osi8*^*1*^ mutants (2-day-old animals). Scale bars, 50 μm. The higher-magnification images (scale bars, 10 μm; samples from 20-day-old animals) highlight several trichoid sensilla on the distal edge of the antenna; the basal drums of these sensilla are indicated with yellow arrowheads. **D** Representative traces of electrophysiological responses of Or47b OSNs to palmitoleic acid (10^−1^ v/v) (0.5 s stimulus, gray bar) in control (*w*^*1118*^), *Osi8* mutant (*Osi8*^*1*^), control rescue (*UAS-CD4:tdTomato/+;Osi8*^*1*^*/Osi8*^*1-DsRed*^*,Osi8-Gal4*), and *Osi8* rescue (*UAS-Osi8/+;Osi8*^*1*^*/Osi8*^*1-DsRed*^*,Osi8-Gal4*) animals. Raster plots and peristimulus time histograms (PSTHs) of these responses are shown below each trace. Line width in the PSTH represents the SEM. **E** Dose-response curves of Or47b OSN responses to palmitoleic acid; genotypes are color-coded as in **D**. Mean responses ±SEM are plotted. *n* = 10 sensilla; 4–5 flies. Statistical comparisons between genotypes were performed by two-way ANOVA: NS *P* > 0.05, *** *P* < 0.001. Full statistical analyses are provided in Additional file 10: Data S2. **F** Representative traces, raster plots, and PSTHs of electrophysiological responses of Or88a OSNs to methyl palmitate (10^−1^ v/v) (0.5 s stimulus, gray bar) in the same genotypes as in **D**. **G** Dose-response curves of Or88a OSN responses to methyl palmitate; genotypes are color-coded as in **D**. Mean responses ±SEM are plotted; *n* = 10 sensilla; 4–5 flies. Statistical analyses were performed as in **E**. **H** Representative traces, raster plots, and PSTHs of electrophysiological responses of Or67d OSNs to *cis*-vaccenyl acetate (10^−1^ v/v) (0.5 s stimulus, gray bar) in control (*w*^*1118*^) and *Osi8* mutant (*Osi8*^*1*^) animals. **I** Dose-response curves of Or67d OSN responses to *cis*-vaccenyl acetate; genotypes are color-coded as in **H**. Mean responses ±SEM are plotted; *n* = 12 sensilla; 4–6 flies. Statistical analyses were performed as in **E**. **J** Basal spiking frequency of the indicated OSNs for control (*w*^*1118*^) and *Osi8* mutant (*Osi8*^*1*^) animals; *n* = 12 sensilla, from 3 flies. *t*-test: NS *P* > 0.05

Analysis of the spatial distribution of *Osi8* RNA FISH signal revealed broad expression around the latero-distal region of the antenna, where trichoid and intermediate sensilla are located (Figs. 2B and 3F; specificity of signal is validated in Fig. 4B), but not in the region where basiconic sensilla are most abundant [11, 12]. Closer examination revealed two types of *Osi8*-expressing cells: one population is distally located in the antenna and has an elongated cell morphology; the other is more proximal, exhibiting a rounder shape and has, generally, a weaker *Osi8* RNA FISH signal (Fig. 3F). To examine whether *Osi8* expression is associated with specific sensillar classes, we visualized *Osi8* RNA simultaneously with GFP transgenic reporters for antennal trichoid (at) and antennal intermediate (ai) sensilla (Fig. 3G). For at1 (labeled with *Or67d*-Gal4-driven mCD8:GFP), the rounder, more weakly labeled *Osi8*-positive cells were observed adjacent to the OSN somas (Fig. 3G). at4 sensilla distribution (labeled with *Or88a*-mCD8:GFP) most closely resembled that of the more elongated, strongly expressing *Osi8* cells, although in this case the support cell bodies were located distally to the neuronal somas (Fig. 3G). The two intermediate sensillar classes—ai2, labeled with *Or83c-*mCD8:GFP, and ai3, labeled with *Or2a*-mCD8:GFP—were intermingled with *Osi8*-positive cells. While we cannot exclude the possibility that *Osi8* is also expressed in support cells in these sensillum types, the lack of a consistent spatial relationship between these sensilla and *Osi8*-positive cells suggests that *Osi8* is expressed in support cells predominantly or exclusively in trichoid sensilla. Consistently, we detected 114 *Osi8*-positive cells per antenna (±4 SD; *n* = 5 antennae), which matches well with the total number of at1 and at4 sensilla (68 and 48, respectively; T. O. Auer and L. Abuin, *personal communication*).

Support cells are named after their developmental roles in construction of sensillar cuticular specializations: trichogen (shaft cell), thecogen (sheath cell), and tormogen (socket cell) (Fig. 3H), although these cells also have functions in mature antennae in secreting high levels of Obps and odorant-degrading enzymes into the sensillum lymph [13, 53]. To determine in which cell type(s) *Osi8* is expressed, we performed *Osi8* RNA FISH on antennae in which different support cells were labeled with transgenic markers. No overlap of *Osi8*-positive cells was observed with a marker of thecogen cells, *nompA-*mCD8:GFP (Fig. 3H). By contrast, both distal and proximal populations of *Osi8*-positive cells co-localized with the tormogen cell marker *ASE5*-mCD8:GFP (Fig. 3H and Additional file 1: Figure S1A). Although there is no known marker for the trichogen cell population in the antenna, these observations are consistent with selective *Osi8* expression in tormogen cells in trichoid sensilla, most likely corresponding to cluster O3 in the Fly Cell Atlas (Fig. 2B, G).

To examine the localization of Osi8 protein in tormogen cells, we generated a GFP-tagged version of Osi8 and expressed this under the control of an *Osi8* promoter-Gal4 driver (Additional file 1: Figure S1B). GFP:Osi8 was detected around the nuclear membrane (presumably the endoplasmic reticulum) and in prominent vesicle-like puncta (Fig. 3I). We also detected GFP within the lumen near the base of the sensillum shaft (Fig. 3I), which may correspond to the occasional protrusions of the tormogen cell and/or extracellular vacuoles that likely derive from this cell type [40]. Confirmation of this localization pattern will require development of specific Osi8 antibodies to detect endogenous protein, but the distribution of GFP:Osi8 in various membranous organelle-like structures is similar to that of other Osi proteins [50, 90, 91].

### Osi8 is required for high sensitivity pheromone-evoked responses

To determine the function of Osi8, we generated a null mutant by CRISPR/Cas9-mediated replacement of the *Osi8* locus with a *DsRed* reporter (which was subsequently removed) (Fig. 4A). Homozygous *Osi8* mutants are viable and fertile, and the absence of *Osi8* transcripts in the antenna was confirmed by RNA FISH (Fig. 4B). Previous work has suggested that Osi proteins have roles in shaping of the cuticle [50, 91]. Notably, in the maxillary palp—a secondary olfactory organ of insects—Osi23 is expressed in developing (but not mature) trichogen cells and is required for the formation of the pores in the sensillar shaft through which odors pass (see “Discussion”) [50]. Scanning electron microscopy of trichoid sensilla of *Osi8* mutant antennae did not reveal any overt morphological defects, including in the basal drum (the rounded base of the sensillum secreted by the tormogen cell) (Fig. 4C). Moreover, there were no noticeable defects in the mechanical flexibility of the *Osi8* mutant trichoid sensilla as assessed qualitatively during electrophysiological recordings (see below). While we cannot exclude the possibility of more subtle cuticular defects, the different spatio-temporal expression pattern of *Osi8* (Fig. 3B) compared to *Osi23* (and other family members) suggests that Osi8 plays a role in the mature function, rather than the development, of these sensilla.

To test this hypothesis, we measured the activity of OSNs within trichoid sensilla through single-sensillum electrophysiological recordings. In at4, the responses of Or47b and Or88a neurons to palmitoleic acid and methyl palmitate—their respective cognate ligands [92, 93]—were markedly reduced in *Osi8* mutants compared to controls (Fig. 4D–G), although weak responses were observed at the higher concentrations tested. We validated this loss-of-function phenotype in two ways: first, depletion of *Osi8* transcripts through RNA interference (RNAi) (Additional file 2: Figure S2A) also led to a decrease in pheromone sensitivity of these neurons (Additional file 2: Figure S2B-E). Second, *Osi8-*Gal4-driven expression of an *Osi8* transgene (but not a negative control *CD4:tdTomato* transgene) was sufficient to restore pheromone sensitivity in *Osi8* mutants to control levels (Fig. 4D–G). By contrast, responses of Or67d neurons to their cognate ligand, (*Z*)-11-octadecenyl acetate (*cis*-vaccenyl acetate), were indistinguishable from controls (Fig. 4H,I). Loss of *Osi8* did not affect the basal firing rate of any of these neuronal populations (Fig. 4J).

We explored the origin of the electrophysiological phenotype in at4 sensilla by examining the expression of a variety of cellular markers for this sensillum class in *Osi8* mutants (Additional file 3: Figure S3). Osi8 cells (labeled by *Osi8-Gal4*) were indistinguishable in appearance (by confocal microscopy) in the absence of *Osi8* to control cells, suggesting that *Osi8* is not required for their developmental specification (Additional file 3: Figure S3A). Similarly, a *Lush-Gal4* driver (which labels several support cell types in at4 (and at1) sensilla [29, 54, 94]) displayed the same expression in controls and *Osi8* mutants (Additional file 3: Figure S3B). Finally, we investigated the expression of Or47b and Or88a using transcriptional reporters, but did not detect any noticeable differences in *Osi8* mutants that could explain the attenuated electrophysiological responses of these neurons (Additional file 3: Figure S3C-D).

Together, these results reveal a selective role for Osi8 in promoting high sensitivity of a subset of pheromone-sensing neurons, while not appearing to affect the gross developmental properties of either the neuronal or support cells of these sensilla. The differential requirement for Osi8 in at4 and at1 may reflect differences in the morphology or function of Osi8-expressing cells in these sensillar classes, or another unique property of the at4 sensilla.

## Discussion

We have performed a comparative transcriptomic screen to identify genes that are differentially expressed between the *D. melanogaster* antennal olfactory subsystems, revealing a striking number and diversity of uncharacterized molecules that might contribute to the subsystems’ distinct properties. As RNA samples were collected from adult antennae, it is possible that many of the identified genes have roles in the mature function, rather than the development, of these subsystems. Future transcriptomic analyses of developing *ato* and *amos* mutant antennal tissue should be an effective way to identify genes that contribute to the different developmental features of these subsystems.

Our comparative transcriptomic dataset has also been complementary to the Fly Cell Atlas [29] in advancing cell type annotation of support cells in the antenna. Many subsystem-specific genes are expressed in non-neuronal cells, which has allowed us to demarcate those that form part of the Or or Ir subsystem. However, in contrast to OSN populations, we found that very few (or no) unique molecular markers exist for specific support cell types across or within individual sensillar classes. The molecular heterogeneity of support cells suggests a substantial degree of functional overlap, at least within the main types of olfactory sensilla.

We have further exploited these transcriptomic data to demonstrate the selective in situ expression of one of the Or subsystem-specific molecules, Osi8, in tormogen support cells in trichoid sensilla. Importantly, loss-of-function genetic analyses demonstrate a requirement for this protein for pheromone-evoked neuronal responses. Amongst insect olfactory sensory signaling pathways, pheromone detection is well-recognized to require accessory proteins beyond the olfactory receptors [13, 51]. In *D. melanogaster*, the best characterized are the Obp Lush [56] and two neuronally expressed proteins, the CD36-related Sensory neuron membrane protein 1 (Snmp1) [94, 95] and the DEG/ENaC sodium channel Pickpocket 25 [96]. One important similarity between Osi8 and these proteins is that their loss can be by-passed at high stimulus concentrations [57, 96, 97], indicating that they are not essential components of the signaling cascade but contribute to the high sensitivity and/or selectivity of pheromone detection. Osi8 differs from these other proteins in its function within support cells, and not as a perireceptor protein (like Lush) or in OSNs (like Snmp1 or Pickpocket 25).

While the mechanism of Osi8 is unknown, some insights may be gained from studies on two other Osi proteins, which have been implicated in regulating membrane trafficking in other tissues. Osi21 controls endolysosomal trafficking in photoreceptor neurons, in which it partially localizes to these organelles [90]. Osi23 localizes, at least in part, to endosomes, and the absence of pores in maxillary palp sensillar hairs in *Osi23* mutants has been traced back to lack of undulations in the plasma membrane of developing trichogen cells, which may prefigure the porous nature of the secreted cuticle layer [50]. Other Osi proteins appear to be expressed in cuticle-secreting cells in various tissues, and some have been detected in vesicular structures [50, 91]. The biochemical function in any case is, however, unclear. Bearing in mind the caveats of transgenic fusion protein expression, the predominant localization of GFP:Osi8 to the endomembrane system of tormogen cells suggests that Osi8 has an analogous role to other Osi proteins in intracellular membrane trafficking. Future ultrastructural and electrophysiological analysis of *Osi8* mutant at4 sensilla will be necessary to determine if this protein contributes to tormogen-specific morphological specializations—such as microvilli and microlamellae bordering the sensillum lymph [40, 41]—and how this might impact the function of pheromone-sensing neurons.

Interestingly, another potential function for Osi8 has emerged from investigations into the molecular basis of octanoic acid resistance of the ecological specialist *Drosophila sechellia*, which feeds uniquely on octanoic acid-rich “noni” fruit [98]. *Osi8* falls within a genomic region identified by unbiased mapping studies as contributing to the tolerance of *D. sechellia* to this acid [99]. Ubiquitous RNAi of *Osi8* in adult or larval *D. melanogaster* led to a decrease in this species’ resistance to octanoic acid exposure [100, 101]. It is unclear in which tissue Osi8 is required for this role, but we suspect that it is unrelated to its function in the antenna, as octanoic acid does not activate neurons in at4 (or at1) sensilla [49, 102]. Our *Osi8* mutant may help validate this RNAi phenotype.

Regardless of its precise function, the conservation of Osi8 across insect taxa—except in *P. humanus*, which exhibits a drastic loss of Ors—together with the antennal expression of the *A. aegypti* ortholog, raise the possibility that Osi8 is a conserved regulator of insect pheromone signaling. More generally, the requirement for Osi8 further highlights the important contribution of proteins in non-neuronal cells for the normal function of associated sensory neurons. While there are recent hints of interactions between support cells and neurons in insect sensilla [103], many of the best-characterized examples are found in *C. elegans*, such as the glial-expressed DEG/ENaC homologs and a chloride channel, which are critical for mechanosensory responses of neighboring nose-touch neurons [104, 105]. Further study of Osi8—as well as the many other non-neuronal genes identified here—should reveal deeper insights into the molecular mechanisms by which specific properties of sensory systems arise from the concerted contributions of neurons and their associated cells.

## Conclusions

Together our work provides a resource for characterization of olfactory subsystem-specific genes, emphasizes the importance of support cells in sensory responses of neurons, and identifies a new protein required for insect pheromone detection.

## Methods

### *D. melanogaster* culture and strains

Animals were grown on standard *Drosophila* culture media at 25°C in a 12-h light:12-h dark cycle. Mutant and transgenic lines used are described in Additional file 6: Table S3. Mixed sexes were used in all experiments, except for the electrophysiology, where only male flies were analyzed.

### Transcriptomic analyses

#### Microarrays

Three biological replicates were analyzed for both *ato*^*1*^*/Df(3R)p13* and *amos*^*3*^. The mutant lines were back-crossed five times with *Oregon-R-P2* prior to the experiment to isogenize the genetic background. Olfactory third antennal segments from ~100–200 adult flies (0–3 days old; mixed sexes (~1:2 males:females—defined by the ratio of *ato* mutants that successfully eclosed—with no selection for virginity) per biological replicate were harvested via snap-freezing animals in a mini-sieve with liquid nitrogen and agitating the animals to break off the appendages [106]. Collected antennae were homogenized manually with a tissue grinder and total RNA was extracted using a standard TRIzol/chloroform protocol, ethanol precipitated, and dissolved to 45 ng/μl final concentration. Samples were hybridized to Affymetrix *Drosophila* Genome 2.0 Arrays. Microarray data—which also include an *Oregon-R-P2* wild-type genotype not presented in this work—are available at the NCBI Gene Expression Omnibus (GSE183763) [107].

#### Antennal RNA-seq

Three biological replicates of control (*w*^*1118*^) and *amos*^*3*^ animals were cultured at 22°C. Antennae were collected from 0–4-h-old adults (mixed sexes) by snap-freezing the animals in dry ice and separating the antennae from other tissues by shaking through a 20-μm sieve. Approximately 300 antennae were transferred into a 1.5-ml Eppendorf tube containing 20 μl Trizol. The antennae were homogenized with an RNase-free pestle until no intact cuticle could be detected and the lysate was transferred into 300 μl Trizol. RNA purification was performed following a standard protocol [108] followed by column purification (RNeasy, QIAGEN). RNA-seq libraries were prepared using the Illumina TruSeq Stranded mRNA protocol; library QC was performed using a Fragment Analyzer (Agilent Technologies). Sequence data was processed using the Illumina Pipeline Software v1.82. Library adapters of purity-filtered reads were trimmed with Skewer v0.1.120 [109] and read quality assessed with FastQC v0.10.1 (Babraham Informatics). Reads were aligned against a *D. melanogaster* reference genome (dmel_r6.02, FlyBase) using TopHat2 v2.0.11 [110]. The numbers of read counts for each *Osi* gene were summarized with HTSeq-count v0.6.1 [111] using *D. melanogaster* (GFF:dmel-all-r6.02, FlyBase) gene annotation. RNA-seq data—which also include similar RNA-seq analyses of third instar larval antennal discs not presented in this work—are available at the NCBI Gene Expression Omnibus (GSE190696) [112].

### New Drosophila mutant and transgenic lines

#### Osi8 mutant

The sgRNA expression vector was generated by PCR amplification of three fragments (encoding four different sgRNAs) using the oligonucleotide pairs listed in Additional file 7: Table S4, and cloning these via Gibson assembly into *BbsI*-digested *pCFD5* (Addgene #73914), as described [113]. The donor vector for homologous recombination was generated by amplifying ~1 kb homology arms (HA) flanking the *Osi8* coding sequence from genomic DNA of *{Act5C-Cas9.P.RFP-}ZH-2A w[*118*]Lig[169]* flies and inserting the products into *pHD-DsRed-attP* (Addgene #51019) via *EcoRI* and *SacII* (HA1) or *SapI* (HA2) restriction cloning. Injection of the sgRNA vector (150 ng μl^−1^) and donor vector (400 ng μl^−1^) into *{Act5C-Cas9.P.RFP-}ZH-2A w[*118*]Lig[169]* flies was performed by BestGene Inc. An *Osi8* mutant (*Osi8*^*1*^) was identified on the basis of DsRed expression and balanced to remove other transgenes; deletion of the *Osi8* gene was validated by PCR (Additional file 7: Table S4). The *Osi8*^*1-DsRed*^ allele was generated by balancing *Osi8*^*1*^ with a transgene encoding the Cre recombinase (Additional file 6: Table S3) and selecting for larvae that had lost the DsRed marker.

#### Osi8-Gal4

3416 bp upstream of the predicted *Osi8* transcription start site (i.e., 100 bp 5′ of the start codon) were PCR amplified (Additional file 7: Table S4), cloned in pCRII-TOPO (Thermo Fisher Scientific), sequenced, and subcloned via *EcoRI/BamHI* sites incorporated into the PCR primers into *EcoRI/BglII* sites of *pGAL4attB* [31]. The construct was inserted into attP2 by phiC31-mediated transgenesis (BestGene Inc.).

#### UAS-Osi8

A genomic region encompassing the *Osi8* coding sequence (with 5′ and 3′ UTRs and intron) was PCR amplified (Additional file 7: Table S4) from Canton-S genomic DNA, cloned into pCR2.1-TOPO and sequenced, before subcloning using *EcoRI* (using the *EcoRI* site incorporated into the forward primer and, at the 3′ end, the *EcoRI* site in pCR2.1-TOPO) into *pUAST-attB* [114]. The construct was inserted into attP40 by phiC31-mediated transgenesis (BestGene Inc.).

#### UAS-SS:EGFP:Osi8 (GFP:Osi8)

The *Osi8* cDNA lacking the first 23 codons (encoding the predicted Osi8 signal sequence) was PCR amplified from Canton-S antennal cDNA (Additional file 7: Table S4), cloned into pCR2.1-TOPO and sequenced, before subcloning using *EcoRI* and *XbaI* into *pUAST-SS:EGFP attB* [26], creating an in-frame fusion with the heterologous signal sequence from calreticulin and the EGFP tag. The construct was inserted into attP40 by phiC31-mediated transgenesis (BestGene Inc.).

### Bioinformatic analyses and other RNA-sequence datasets

Gene ontology term enrichment analyses were performed using GOrilla [68], using genes displaying a >2-fold expression difference between *ato* and *amos*; the Affymetrix *Drosophila* Genome 2.0 Array gene list was used as the reference dataset. Subsequently, genes displaying >4-fold expression difference between these genotypes were curated manually, using annotations from FlyBase [115] and additional analyses using SMART [116] and BLAST [117], the latter usually to verify the absence of similarities to protein domains of known function. *D. melanogaster* antennal and head scRNA-seq data were from the 10× stringent dataset from the Fly Cell Atlas [29]; these were visualized in the HVG tSNE coordinate representation in the SCope interface [118]. Expression data across tissues/life stages for *Osi8* were from Fly Atlas 2 [88]. Expression data of the *Aedes aegypti Osi8* ortholog (AAEL004275, AaegL3.3 annotation) were from a published dataset [89].

### Histology

RNA FISH (based upon the Tyramide Signal Amplification™ method (Perkin Elmer)) and protein immunofluorescence on whole-mount antennae or antennal cryosections were performed essentially as described [106], using 1–10-day-old animals. Templates for RNA FISH probes were amplified by PCR from genomic DNA (Additional file 7: Table S4) and cloned into pGEM-T Easy (Promega) or pCRII-TOPO; the *Osi8* probe was synthesized from the same genomic region used to construct the *UAS-Osi8* transgene. Antibodies used are listed in Additional file 8: Table S5. Imaging was performed on a Zeiss LSM710 or LSM880 Airyscan confocal microscope using 40× or 63× oil immersion objectives. Confocal stacks were imported into Fiji [119] for processing and analysis and subsequently formatted in Adobe Photoshop 2022. Cell counting was performed manually using the Cell Counter plugin of ImageJ. For each experiment, the expression/phenotype was assessed in antennae from at least 10 animals in at least two independent replicates. Experimenters were not blind to the gene or genotype under assessment.

### Electron microscopy

Control (*w*^*1118*^) and *Osi8*^*1*^ 2-day or 20-day-old animals were fixed in 2.5% glutaraldehyde solution in 0.1 M phosphate buffer pH 7.4 (PB) for 2 h at room temperature (RT). Samples were rinsed 3 × 5 min in PB and post-fixed in a fresh mixture of 1% osmium tetroxide (EMS) with 1.5% potassium ferrocyanide in PB for 2 h at RT. After washing twice in distilled water, samples were dehydrated in acetone solution at graded concentrations (30% (40 min), 50% (40 min), 70% (40 min), 100% (3 × 1 h)). Samples were dried at the critical point (BALTEC CPD 30) and glued on a pin and sputter coated with 10 nm of metal platinum (LEICA EM SCD 500). Micrographs were taken with a scanning electron microscope FEI Quanta FEG 250 (FEI, Eindhoven, the Netherlands) with an Everhart-Thornley Detector (ETD).

### Electrophysiology

For all electrophysiological experiments, 7-day-old male flies (housed in groups of 10) were used. An animal was prepared for recordings by wedging it into the narrow end of a truncated 200-μl plastic pipette tip to expose the antennae, one of which was then stabilized between a tapered glass microcapillary tube and a coverslip covered with double-sided tape. Single-unit recordings were performed essentially as described [120]. In brief, the electrical activity of the neurons was recorded extracellularly by inserting a sharp electrode filled with artificial hemolymph solution [121] into either an at4 sensillum (Or47b and Or88a OSN recordings) or an at1 sensillum (Or67d OSN recording). A reference electrode filled with the same solution was inserted into the eye. Odor stimuli: *trans*-palmitoleic acid (Cayman Chemical, CAS 10030-73-6) and *cis*-vaccenyl acetate (Cayman Chemical, CAS 6186-98-7) were diluted in ethanol, and methyl palmitate (Sigma-Aldrich, CAS 112-39-0) was diluted in paraffin oil. 4.5 μl aliquots of dilutions were spotted onto filter discs and delivered via a 500-ms air pulse at 250 ml/min directly to the antenna from close range, as described [120]. Ethanol was allowed to evaporate before the experiments. Spike responses were averaged, binned at 50 ms, and smoothed using a binomial algorithm to obtain peristimulus time histograms (PSTHs). For dose-response curves and statistical analysis, responses were quantified by subtracting the pre-stimulus spike rate (1 s) from the peak spike response during odorant stimulation (adjusted peak responses). Basal neuronal activity was measured during 10 s from flies that had not been exposed to odor stimuli. Electrophysiological data are provided in Additional file 10: Data S2.

## Supplementary Information

**Additional file 1: Figure S1. ***Osi8* tormogen cell expression and validation of *Osi8-Gal4*. (A) GFP immunofluorescence and *Osi8* RNA FISH on a whole-mount antenna in which the tormogen support cells are labeled (*UAS-mCD8:GFP;ASE5-Gal4*). The white arrowheads point to examples of proximally located cells expressing both *Osi8* and GFP. Scale bar, 20 μm. (B) GFP immunofluorescence and *Osi8* RNA FISH on antennal cryosections of *UAS-mCD8:GFP/+;Osi8-Gal4/+* animals. Scale bar, 20 μm. The white arrowheads point to examples of cells expressing GFP where *Osi8* transcripts are not detected. Of 176 cells analyzed in four antennae, 135 (77%) express both *Osi8* RNA and GFP, while 41 (23%) express only GFP. The latter category may be due to the lower sensitivity of RNA FISH and/or ectopic *Osi8-Gal4* expression. Note also the heterogeneous (and often uncorrelated) expression level of *Osi8* RNA and GFP.

**Additional file 2: Figure S2.** Phenotypic analysis of *Osi8* RNAi. (A) *Osi8* RNA FISH on control (*Act5C-Gal4,UAS-Dcr-2/+*) and *Osi8*^*RNAi*^ (*UAS-Osi8*^*RNAi*^/+;*Act5C-Gal4,UAS-Dcr-2/+*) whole-mount antennae. Scale bar, 20 μm. (B) Representative traces of electrophysiological responses of Or47b OSNs to palmitoleic acid (10^-1^ v/v) (0.5 s stimulus, gray bar) in *Gal4* control (*Act5C-Gal4,UAS-Dcr-2/+*), *UAS* control (*UAS-Osi8*^*RNAi*^/+) and *Osi8*^*RNAi*^ (*UAS-Osi8*^*RNAi*^/+; *Act5C-Gal4,UAS-Dcr-2/+*) animals. Raster plots and PSTHs of these responses are shown below each trace. (C) Dose-response curves of Or47b OSN responses to palmitoleic acid; genotypes are color-coded as in (B). Mean responses ±SEM are plotted; n = 12 sensilla; 4-6 flies. Statistical comparisons between genotypes were performed by two-way ANOVA: NS *P* > 0.05, *** *P* < 0.001. Full statistical analyses are provided in Additional file 10: Data S2. (D) Representative traces, raster plots and PSTHs of electrophysiological responses of Or88a OSNs to methyl palmitate (10^-1^ v/v) (0.5 s stimulus, gray bar) in the same genotypes as in (B). (E) Dose-response curves of Or88a OSN responses to methyl palmitate; genotypes are color-coded as in (B). Mean responses ±SEM are plotted; n = 12 sensilla; 4-6 flies. Statistical analyses were performed as in (C).

**Additional file 3: Figure S3.** Cellular phenotypic characterization of *Osi8* mutants. (A) GFP immunofluorescence labeling Osi8 cells in control (*UAS-mCD8:GFP/+;Osi8-Gal4/+*) and *Osi8* mutant (*UAS-mCD8:GFP/+;Osi8-Gal4,Osi8*^*1*^*/Osi8*^*1*^) antennae. Scale bar, 20 μm. (B) GFP immunofluorescence labeling Lush cells in control (*Lush-Gal4/UAS-mCD8:GFP*) and *Osi8* mutant (*Lush-Gal4/UAS-mCD8:GFP;Osi8*^*1*^) antennae. Scale bar, 20 μm. (C) GFP immunofluorescence labeling Or47b neurons in control (*Or47b-Gal4/UAS-mCD8:GFP*) and *Osi8* mutant (*Or47b-Gal4/UAS-mCD8:GFP;Osi8*^*1*^) antennae. Scale bar, 20 μm. (D) GFP immunofluorescence labeling Or88a neurons in control (*Or88a-mCD8:GFP/+*) and *Osi8* mutant (*Or88a-mCD8:GFP/+;Osi8*^*1*^) antennae. Scale bar, 20 μm.

**Additional file 4: Table S1.** Analysis of Ir and Or subsystem-enriched genes. Annotations of genes enriched in the Ir (magenta) and Or (green) subsystems, as defined as those showing a >4-fold differential expression between *amos* and *ato* mutant antennae. This arbitrary cut-off captures all of the known chemosensory receptors expressed in the two subsystems. The dataset was cleaned from the original microarray data (GSE183763) by updating gene names and removing duplicate gene entries and those corresponding to transposons. Neuronal/non-neuronal expression is a qualitative assessment based upon the Fly Cell Atlas antennal dataset [29] and/or RNA FISH data for chemosensory receptors [17–19]: “1” = expressed; “0” = not expressed; empty cells indicate robust expression was not detected in either cell type (which may reflect sub-threshold expression level). Breadth of expression provides an approximate categorization of cell-type specific and ubiquitously expressed genes within each olfactory subsystem, based upon the qualitative assessment of the number of clusters in the Fly Cell Atlas antennal dataset in which a gene is detected: “1” = 1 cluster; “2” = 2-5 clusters; “3” = >5 clusters.

**Additional file 5: Table S2.** Definition of Ir and Or subsystem support cell populations. Populations were numbered arbitrarily, based upon the Fly Cell Atlas antennal cell cluster representation [29]; it is likely that they can be subclustered further. The “I” and “O” categorization was defined post-hoc, based upon a qualitative assessment of their preferential expression of Ir subsystem- or Or subsystem-enriched genes (from Additional file 4: Table S1; undetected genes were excluded). The I12 and I13 clusters express *Obp84a*, which is thought to be expressed in thecogen cells based upon exclusive co-expression with a *nompA* promoter transgenic reporter [54]. Endogenous *nompA* transcripts are detected consistently (albeit weakly) in I12 but only very sparsely in I13.

**Additional file 6: Table S3. ***D. melanogaster* strains [122–124].

**Additional file 7: Table S4.** Oligonucleotides.

**Additional file 8: Table S5.** Antibodies.

**Additional file 9: Data S1.** Gene ontology (GO) term analyses of Or and Ir subsystem-enriched genes. Enriched GO terms for Or subsystem genes (>2-fold more highly expressed in *ato* versus *amos* antennae from the microarray analysis) and Ir subsystem genes (>2-fold more highly expressed in *amos* versus *ato* antennae), organized by ontology (Process, Function, Component) on different worksheets. Data represent the output from GOrilla [68], including the GO term and gene lists, enrichment terms and statistical analyses (see the bottom of the first worksheet for the definitions of values). Below each table is the graphical visualization of the hierarchy of enriched GO terms (from GOrilla), to emphasize the statistically significant, enriched nodes.

**Additional file 10: Data S2.** Electrophysiological quantifications. Raw spike counts, processed data and statistical comparisons are shown for all electrophysiological experiments, organized by figure panel on different worksheets.

## Data Availability

All data generated or analyzed during this study are included in this published article, its supplementary information files (Additional file 10: Data S2) and publicly available repositories: NCBI Gene Expression Omnibus GSE183763 (comparative antennal microarray data) and GSE190696 (bulk antennal RNA-seq data), SCope (Fly Cell Atlas data), and FlyAtlas 2 (tissue-specific bulk RNA-seq data).
