# Supplementary material for: Pheromone sensing in Drosophila requires support cell-expressed Osiris 8
Source: BMC Biol. 2022 Oct 11;20:230. doi: 10.1186/s12915-022-01425-w (PMC9552441; doi:10.1186/s12915-022-01425-w)
Supplement: Supplementary file 6 — Additional file 6: Table S3. D. melanogaster strains [122–124]. [file 12915_2022_1425_MOESM6_ESM.pdf]

Additional file 6: Table S3. *D. melanogaster* strains.

Genotype	Reference
Oregon-R-P2	RRID:BDSC_2376
w^{1118}	RRID:BDSC_3605
Canton-S	RRID:BDSC_64349
ato^{1}	[122]; RRID:BDSC_25779
Df(3R)p13	RRID:BDSC_1943
$amos^{3}$	[29]
UAS-mCD8:GFP	RRID:BDSC_5137
UAS-CD4:tdTomato	RRID:BDSC_35841
$Or67d^{Gal4\#1}$	[123]
Or88a-mCD8:GFP	RRID:BDSC_52644
Or83c-mCD8:GFP	RRID:BDSC_52639
Or2a-mCD8:GFP	RRID:BDSC_52611
Or47b-Gal4	RRID:BDSC_9983
nompA-Gal4	[50, 124]
ASE5-Gal4	RRID:BDSC_93029
lush-Gal4	[88]
Cre (recombinase)	RRID:BDSC_851
{Act5C-Cas9.P.RFP-}ZH-2A w[118] Lig[169]	RRID:BDSC_58492
$Osi8^{1}$	*This work*
$Osi8^{1\text{-}DsRed}$	*This work*
Osi8-Gal4	*This work*
UAS-Osi8	*This work*
UAS-SS:EGFP:Osi8	*This work*
Act5C-Gal4,UAS-Dcr-2	RRID:BDSC_3954, RRID:BDSC_24651
Osi8-15591R-1 ($UAS\text{-}Osi8^{RNAi}$) (chr. II)	NIG-FLY
