# Supplementary material for: Pheromone sensing in Drosophila requires support cell-expressed Osiris 8
Source: BMC Biol. 2022 Oct 11;20:230. doi: 10.1186/s12915-022-01425-w (PMC9552441; doi:10.1186/s12915-022-01425-w)
Supplement: Supplementary file 7 — Additional file 7: Table S4. Oligonucleotides. [file 12915_2022_1425_MOESM7_ESM.pdf]

Additional file 7: Table S4. Oligonucleotides.

Name	Use	Sequence
sgRNA1/2f	*Osi8* sgRNA vector	GCGGCCCGGGTTCGATTCCCGGCCGATGCAGATCAAGTACGTGTGGCATGGTTTTAGAGCTAGAAATAGCAAG
sgRNA1/2r	*Osi8* sgRNA vector	ACAGCGGCTGGAAGCCGGCGTGCACCAGCCGGGAATCGAACCC
sgRNA2/3f	*Osi8* sgRNA vector	CGCCGGCTTCCAGCCGCTGTGTTTTAGAGCTAGAAATAGCAAG
sgRNA2/3r	*Osi8* sgRNA vector	AACAGTCTGAGCTCGGCCCCTGCACCAGCCGGGAATCGAACCC
sgRNA3/4f	*Osi8* sgRNA vector	GGGGCCGAGCTCAGACTGTTGTTTTAGAGCTAGAAATAGCAAG
sgRNA3/4r	*Osi8* sgRNA vector	ATTTTAACTTGCTATTTCTAGCTCTAAAACGACCCCAGCCGGAATGTCCTTGCACCAGCCGGGAATCGAACCC
HA1f	*Osi8* donor vector	CGCCGAATTCGGATCAATAGCCTA
HA1r	*Osi8* donor vector	GCGGCCGCGGGACTGTGCTGGGTA
HA2f	*Osi8* donor vector	GGCCGCTCTTCGTATGCTCGCATTTGCATTTGGAA
HA2r	*Osi8* donor vector	GCGCGCTCTTCGGACTACATGCCTCTGCCTCTGGT
$Osi8^1f$	*Osi8¹* validation	AATTCATCAAGTACGTGTGGCAT
$Osi8^1r$	*Osi8¹* validation	CTCGAGCAGGCTCTTGGCGCTGCC
$Osi8^1$-HA1f	*Osi8¹* validation (control)	CGCCGAATTCGGATCAATAGCCTA
$Osi8^1$-HA1r	*Osi8¹* validation (control)	GCGGCCGCGGGACTGTGCTGGGTA
Osi8promf	*Osi8-Gal4*	GGATCCTCCAAAGACGAAAACTGG
Osi8promr	*Osi8-Gal4*	GAATTCCTTTGGGTGCTCAACTGA
Osi8f	*UAS-Osi8*	GAATTCAGAAGCGCCCGGCGTCAT
Osi8r	*UAS-Osi8*	TCTAGACGGGTAGCAGAATTTACT
Osi8-SSf	*UAS-SS:EGFP:Osi8*	GAATTCGCCAGCTACCAGCATCAG
Osi8r	*UAS-SS:EGFP:Osi8*	TCTAGATCACAGGCTCTTGGCGCT
GstE4f	RNA FISH probe	TATCGCTATACGGCCTGGAC
GstE4r	RNA FISH probe	CCTCAAGAGCTCCACGAACT
Tsp47Ff	RNA FISH probe	GTTCTTGCGGTCCGAGTTTA
Tsp47Fr	RNA FISH probe	GATCAGGATGATGGCGTTCT
dvef	RNA FISH probe	ACACCGAGGACTTGAACACC

dver	RNA FISH probe	GGAGGGGCTCTCAAATAAGG
CG13285f	RNA FISH probe	CCGCCGTATTCAGATTGTTT
CG13285r	RNA FISH probe	GATTCCGCTGACCCTGATAA
CG34456f	RNA FISH probe	CAACAAGTGGCTATATAAGATTGCAG
CG34456r	RNA FISH probe	TTTGTTTTGATAAATTGAAAGAAAGC
CG14153f	RNA FISH probe	GCACTTATGCCCCTGATGAT
CG14153r	RNA FISH probe	AATGGCTCTTGAGGGGATTT
a10f	RNA FISH probe	ATCGATTCGAAAATGGGACA
a10r	RNA FISH probe	TTGGCCTTGGACTTCATCTC
a5f	RNA FISH probe	AGCCAGGATAACGACGAGAA
a5r	RNA FISH probe	CCCATTGCATTAGCAATCCT
CG10357f	RNA FISH probe	ACATGCCGCTTTGCTAGACT
CG10357r	RNA FISH probe	GGGGTTTAACCGCCATCTAT
Jhedupf	RNA FISH probe	AGCTGTTGCTGTCGGAGATT
Jhedupr	RNA FISH probe	TTTTGGATCTCCTCCGAATG
Obp59af	RNA FISH probe	CAACGATGGAGGTCAGTGTG
Obp59ar	RNA FISH probe	CACAAACCATTCAACGCAAC
