# Supplementary material for: Pheromone sensing in Drosophila requires support cell-expressed Osiris 8
Source: BMC Biol. 2022 Oct 11;20:230. doi: 10.1186/s12915-022-01425-w (PMC9552441; doi:10.1186/s12915-022-01425-w)
Supplement: Supplementary file 8 — Additional file 8: Table S5. Antibodies. [file 12915_2022_1425_MOESM8_ESM.pdf]

Additional file 8: Table S5. Antibodies.

Antibody	Dilution	Reference/source	Identifier
anti-DIG-POD	1:300	Roche Diagnostics AG	11 207 733 910
anti-Fluorescein-POD	1:300	Roche Diagnostics AG	11 426 346 910
rabbit-anti-ORCO	1:200	[24]	
guinea pig-anti IR8a	1:300	[26]	RRID:AB_2566833
rabbit anti-GFP	1:1000	Invitrogen (Molecular Probes)	A-6455
chicken anti-GFP	1:1000	Abcam	ab13970
Alexa Fluor 488 goat anti-guinea pig	1:1000	Invitrogen (Molecular Probes)	A11073
Alexa Fluor 488 goat anti-rabbit	1:100	Invitrogen (Molecular Probes)	A11034
Alexa Fluor 488 goat anti-chicken	1:1000	Abcam	ab150169
